# Phenotypic and genomic characterization of linezolid non-susceptibility in poultry-derived multidrug-resistant *Enterococcus* spp. from Hungary

**DOI:** 10.3389/fvets.2026.1781468

**Published:** 2026-05-19

**Authors:** Ádám Kerek, Levente Radnai, Gergely Tornyos, Krisztián Bányai, Eszter Kaszab, Ákos Jerzsele

**Affiliations:** 1 Department of Pharmacology and Toxicology, University of Veterinary Medicine Budapest, Budapest, Hungary; 2 National Laboratory of Infectious Animal Diseases, Antimicrobial Resistance, Veterinary Public Health and Food Chain Safety, University of Veterinary Medicine Budapest, Budapest, Hungary; 3 Department of Medical Biology, Medical School, University of Pécs, Pécs, Hungary; 4 HUN-REN Veterinary Medical Research Institute, Budapest, Hungary; 5 One Health Institute, University of Debrecen, Debrecen, Hungary; 6 Department of Microbiology and Infectious Diseases, University of Veterinary Medicine, Budapest, Hungary

**Keywords:** antimicrobial resistance, *Enterococcus*, linezolid, multidrug resistance, One Health, poultry, whole-genome sequencing

## Abstract

**Background:**

Linezolid is a critically important oxazolidinone in human medicine, and the emergence of linezolid non-susceptibility in food-animal–associated enterococci raises One Health concerns across the food chain, animal reservoirs, and environmental circulation.

**Methods:**

We analyzed poultry-derived *Enterococcus* spp. from healthy broiler chicken and turkey farms across seven Hungarian regions. From a total collection of 969 isolates, a resistance-enriched analytical subset (*n* = 218) underwent broth microdilution testing; linezolid results were interpreted transparently using both clinical breakpoints (CLSI, Clinical and Laboratory Standards Institute) and epidemiological wild-type/non-wild-type classification (EUCAST, European Committee on Antimicrobial Susceptibility Testing), acknowledging their distinct intent and scope. Whole-genome sequencing was performed on a targeted subset (*n* = 73) enriched for elevated or borderline linezolid minimum inhibitory concentrations to screen for transferable oxazolidinone resistance determinants and target-site variation.

**Results:**

Linezolid minimum inhibitory concentrations ranged from 0.015 to 512 μg/mL, with a MIC_50_ = 2 μg/mL and a 90th percentile (MIC_90_) of 128 μg/mL. By clinical categorization, 139/218 (63.7%) isolates were susceptible, 37/218 (17.0%) intermediate, and 42/218 (19.3%) resistant, yielding 79/218 (36.2%) linezolid non-susceptible isolates in this enrichment-based dataset (not designed for prevalence inference). In the sequenced subset, we did not detect the major transferable oxazolidinone resistance genes *optrA*, *poxtA*, or *cfr/cfr*-like, while a 23S rRNA target-site substitution (G2576T) was identified in seven isolates, underscoring heterogeneous and sometimes non-transferable genomic correlates of elevated phenotypes.

**Conclusion:**

Integrating phenotypic distributions with genome-informed screening provides a robust One Health framework to characterize linezolid non-susceptibility in poultry-derived multidrug-resistant *Enterococcus*, while highlighting the need for cautious inference under enrichment-based designs and for surveillance approaches that capture both canonical and under-resolved mechanisms.

## 1 Introduction

Antimicrobial resistance (AMR) has become a defining constraint of modern infectious disease control, eroding the effectiveness of routine therapy and amplifying the downstream risks of invasive procedures, intensive care, and immunosuppressive treatments (1–4). Because the selective and transmission landscapes of resistant bacteria span humans, animals, food systems, and the environment, AMR is intrinsically a One Health problem rather than a sector-specific phenomenon (5, 6). Food-producing animals can serve as reservoirs and mixing vessels for resistant bacteria and resistance determinants, with opportunities for dissemination along the food chain and through environmental interfaces (7–9). Among the organisms positioned at this human–animal–environment nexus, *Enterococcus* spp. are particularly relevant: they are ubiquitous in animal and human gastrointestinal tracts, persist in diverse environmental matrices, and combine ecological fitness with a substantial capacity for genetic adaptation (10–12).

The public health importance of *Enterococcus* extends beyond its commensal ecology. *Enterococcus faecalis* and *Enterococcus faecium* are prominent opportunistic pathogens, with *E. faecium* in particular exhibiting high levels of intrinsic and acquired resistance and a pronounced ability to thrive under hospital selection pressures (13, 14). Multiple traits facilitate persistence and spread, including robust colonization potential and biofilm-associated phenotypes that can contribute to survival on surfaces and within host niches (15). Resistance can be mediated by a spectrum of mechanisms, including target alterations, mobile genetic elements, and efflux systems (13, 16). Clinically, vancomycin-resistant enterococci (VRE) remain a major concern, with well-characterized regulatory and genetic architectures underpinning vancomycin resistance phenotypes (17, 18). Surveillance data across Europe underscore the continuing burden and heterogeneity of VRE, highlighting the need for sustained monitoring and refined risk assessment frameworks that account for both clinical and non-clinical reservoirs (19, 20).

In this setting, oxazolidinones—most notably linezolid—occupy a critical role in the treatment of serious Gram-positive infections, including those caused by multidrug-resistant enterococci (21, 22). Importantly, linezolid is classified within the World Health Organization (WHO) AWaRe framework as a “Reserve” antibiotic, reflecting its stewardship-sensitive status and prioritization as a last-resort option against multidrug-resistant organisms (23). The molecular basis of linezolid resistance in enterococci is multifaceted. Classical routes involve mutations in the 23S rRNA target site and, in some contexts, alterations in ribosomal proteins, whereas transferable mechanisms include acquired genes such as *cfr* and the more recently described oxazolidinone/phenicol resistance determinants *optrA* and *poxtA* (21, 22, 24–26). The emergence and dissemination of transferable oxazolidinone resistance is particularly concerning because it creates a plausible pathway for rapid spread across lineages and settings, potentially decoupling resistance risk from local clonal expansion alone (25).

Evidence increasingly supports a One Health relevance for oxazolidinone resistance determinants: *optrA* and *poxtA* have been described in enterococci from livestock-associated contexts and along food-chain–adjacent interfaces, including slaughterhouse-related environments and retail poultry meat, underscoring the potential for non-human reservoirs to contribute to the broader resistome (27–29). At the same time, interpreting elevated or borderline linezolid minimum inhibitory concentrations (MICs) in animal-derived enterococci is not always straightforward, because the meaning of phenotypic categorization depends on whether one applies clinical breakpoints (treatment-oriented categories) or epidemiological cut-off values (ECOFFs) intended to separate wild-type from non-wild-type populations (30). This distinction matters: non-wild-type status signals acquired or mutational deviation from the baseline population, but it does not automatically imply clinical failure, whereas clinical categories are anchored to dosing, pharmacokinetics/pharmacodynamics, and outcome considerations (30, 31).

Against this background, poultry production represents a strategically important surveillance domain. Poultry systems are high-throughput, geographically distributed, and closely connected to food supply chains, creating repeated opportunities for selection and dissemination of resistant commensals (7, 8). Moreover, *Enterococcus* spp. are frequent indicators in both animal and environmental microbiology, making them useful sentinels for tracking resistance dynamics that may later intersect with clinical populations (7, 10, 11). Recent work has reinforced that poultry-associated enterococci can harbor multidrug resistance phenotypes, motivating targeted investigations that combine rigorous phenotypic measurement with genome-resolved characterization (26, 32, 33).

**Figure 1:**
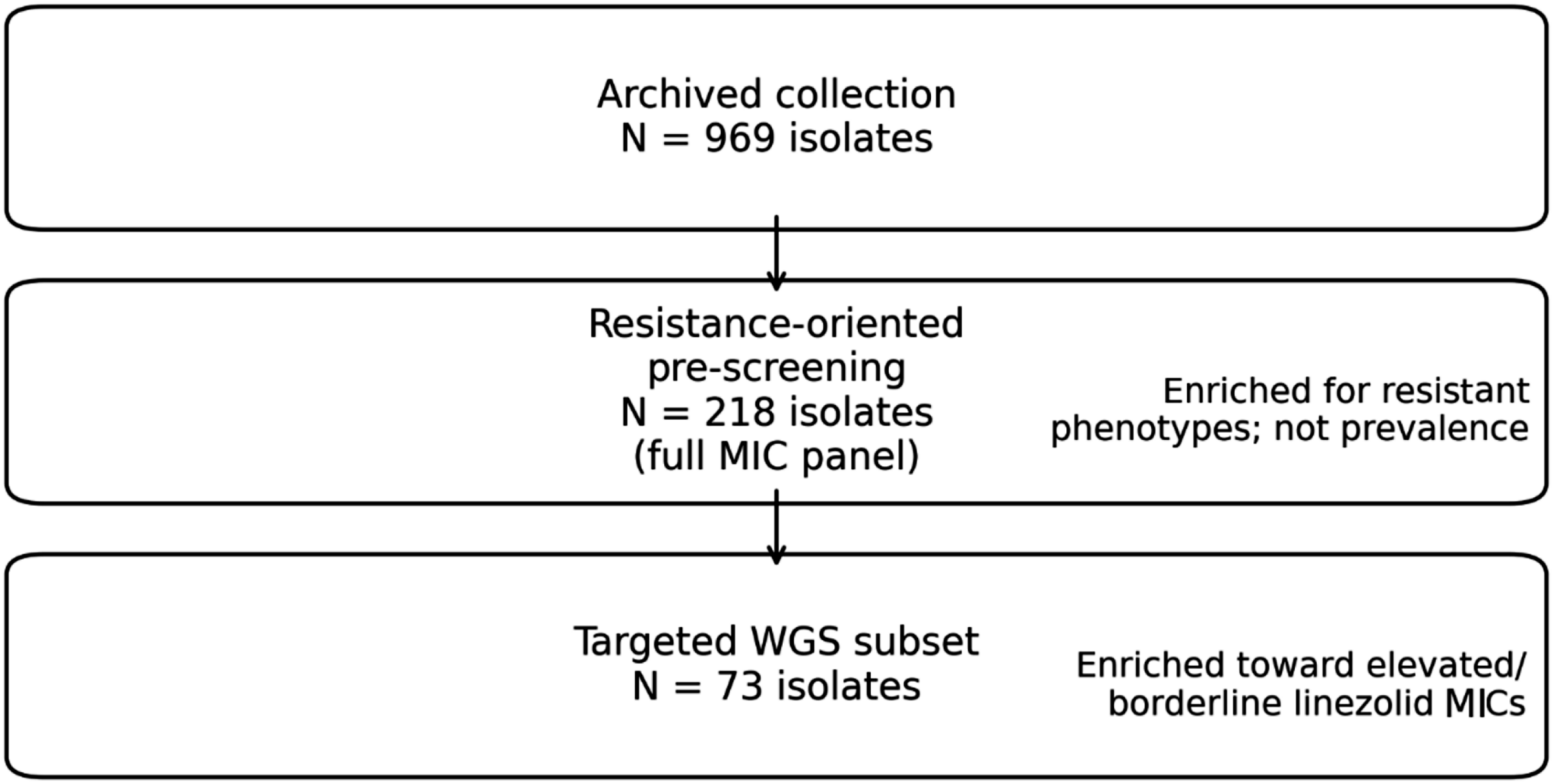
Study flow diagram. Overview of isolate inclusion from the archived collection (*N* = 969) through resistance-oriented selection for expanded minimum inhibitory concentration (MIC) testing (*n* = 218) and targeted whole-genome sequencing (*n* = 73). Both downstream subsets were enrichment-based and are not intended for prevalence inference.

Here, we integrate standardized phenotypic susceptibility testing with whole-genome sequencing to characterize linezolid non-susceptibility patterns and their genetic correlates in poultry-derived multidrug-resistant *Enterococcus* spp. from Hungary. Building explicitly on a One Health framing (6), the study is designed to (i) quantify and visualize linezolid MIC distributions within an enriched multidrug-resistant subset, (ii) interpret phenotypes transparently using clinically oriented categories alongside wild-type/non-wild-type concepts where applicable, and (iii) map known oxazolidinone resistance determinants and relevant target-site variation using high-confidence genomic criteria. By aligning phenotypic and genomic evidence without overextending inferential claims about national prevalence or validated transfer, this work aims to provide a robust contribution that is directly actionable for surveillance, risk assessment, and stewardship across the human–animal interface (Figure 1).

## 2 Materials and methods

### 2.1 Study design, isolate collection, and enrichment strategy

Between 2022 and 2023, samples were obtained from clinically healthy, large-scale poultry farms in Hungary (broiler chickens and turkeys) across seven regions as part of routine surveillance/diagnostic workflows that preceded the present analyses. The current study is a retrospective analysis performed exclusively on archived bacterial isolates and associated metadata from this previously established collection. Tracheal and cloacal swabs had been collected using Amies-type transport medium without charcoal (Biolab Zrt., Budapest, Hungary), and host species and sampling site (trachea vs. cloaca) were recorded. Swabs were streaked onto selective enterococcal medium (m-*Enterococcus* modified agar; Merck KGaA, Darmstadt, Germany) and incubated at 41 °C for 18–24 h. Presumptive enterococci were subcultured to purity and preserved using the Microbank system (Pro-Lab Diagnostics, United Kingdom) at −80 °C.

**Figure 2:**
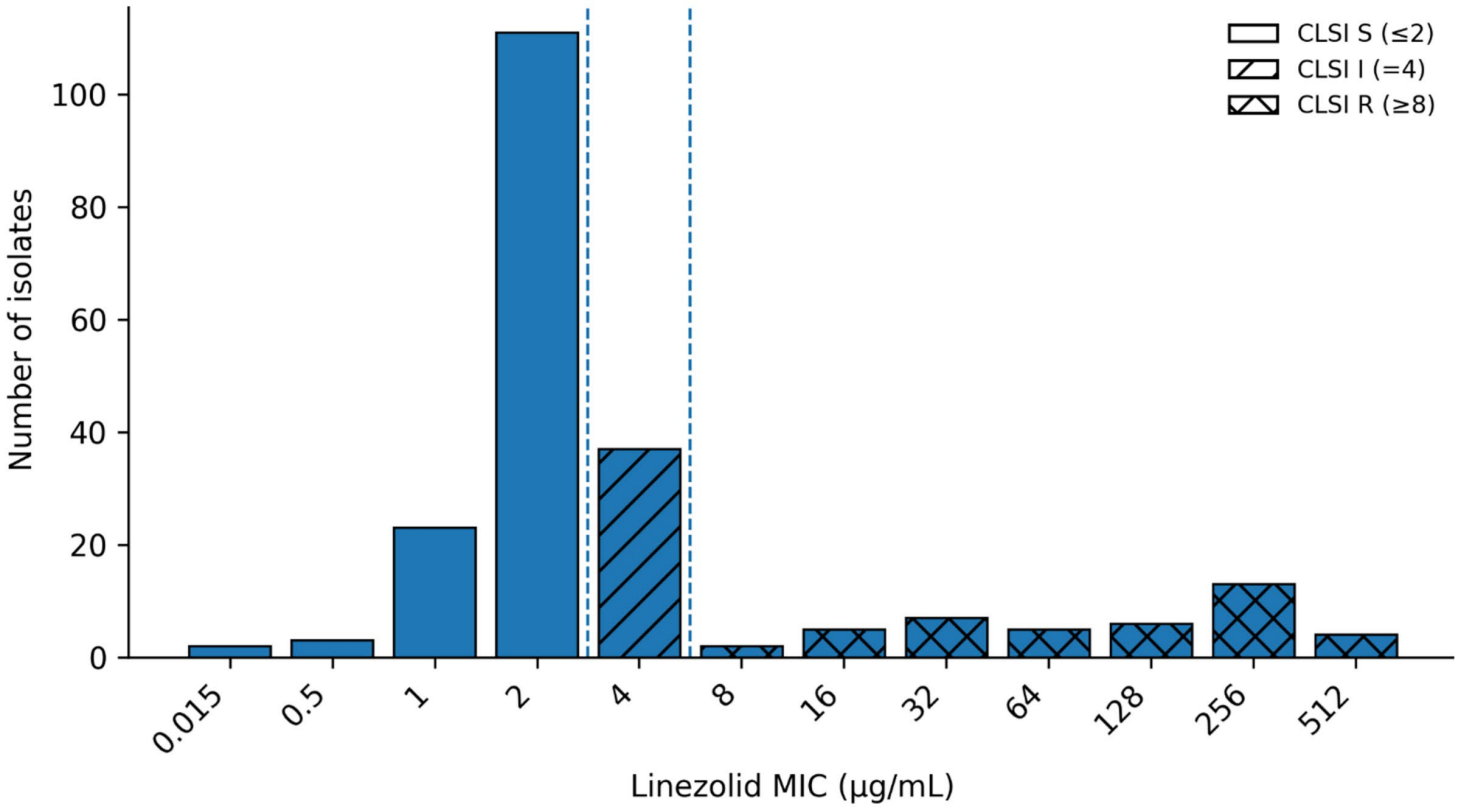
Linezolid minimum inhibitory concentration (MIC) distribution in poultry-derived *Enterococcus* spp. (*n* = 218). Bars show the number of isolates at each broth microdilution MIC value. Colors indicate Clinical and Laboratory Standards Institute (CLSI) clinical categories [susceptible (S), intermediate (I), resistant (R); breakpoints: S ≤ 2 μg/mL, I = 4 μg/mL, R ≥ 8 μg/mL]. The analyzed set represents a resistance-enriched subset and is not intended for prevalence inference.

Resistance-oriented pre-screening and selection of the phenotypic panel (*n* = 218). The archived collection comprised 969 presumptive *Enterococcus* isolates. To enable mechanistic interrogation of multi-class resistance under a feasible workload, we applied a resistance-oriented pre-screening strategy using indicator antimicrobials spanning major *Enterococcus*-relevant classes. The indicator pre-screening panel covered the same major *Enterococcus*-relevant classes represented in the expanded MIC panel, including *β*-lactams (e.g., amoxicillin), glycopeptides (vancomycin), oxazolidinones (linezolid), macrolides (e.g., tylosin, tilmicosin, azithromycin), tetracyclines (doxycycline, oxytetracycline), phenicols (florfenicol), lincosamides (lincomycin), pleuromutilins (tiamulin), and fluoroquinolones (enrofloxacin). The full antimicrobial list and concentration ranges are provided in Supplementary Table S1. Isolates were retained for the expanded broth microdilution panel if they met at least one of the following criteria: non-susceptibility to at least one agent in ≥3 antimicrobial classes represented in the indicator panel; elevated/borderline MICs for high-priority agents relevant to rare high-priority phenotypes (including linezolid, and—phenotypically—vancomycin) to enrich rare phenotypes; and/or representation across poultry host species and geographic regions to avoid redundant sampling from the same source. Importantly, selection was not conditioned on growth at a single linezolid concentration; the final phenotypic panel includes linezolid-susceptible isolates and spans MICs from 0.015 to 512 μg/mL (Figure 2).

Presumptive enterococci were identified by MALDI-TOF MS using a Bruker Microflex platform and the MBT Compass Library (DB-12827). The *α*-cyano-4-hydroxycinnamic acid (HCCA) matrix was applied following the manufacturer’s recommendations. For isolates yielding suboptimal identification scores by direct transfer, on-plate formic acid extraction was performed to improve protein extraction prior to matrix overlay. Identification was accepted at manufacturer-recommended score thresholds for genus- and species-level assignment.

### 2.2 Antimicrobial susceptibility testing and interpretation framework

For MIC testing, isolates were recovered from frozen stocks and grown overnight. Inocula were prepared from fresh colonies and standardized to 0.5 McFarland using a nephelometer, then diluted in cation-adjusted Mueller–Hinton broth (CAMHB). MIC testing was performed using an in-house prepared broth microdilution format with two-fold serial dilutions in sterile 96-well microtiter plates, following ISO 20776-1 principles and CLSI M07 procedural elements (inoculum preparation, incubation, and endpoint reading). The final inoculum was approximately 5 × 10^5 CFU/mL in cation-adjusted Mueller–Hinton broth, plates were incubated at 37 °C for 18–24 h, and MICs were read as the lowest concentration with complete inhibition of visible growth. For bacteriostatic agents where trailing growth can occur (including linezolid), MIC endpoints were read in accordance with CLSI/EUCAST broth microdilution reading guidance, ignoring faint trailing/haze and using the first well with clearly inhibited growth as the endpoint; borderline results were re-checked in independent runs. *Enterococcus faecalis* ATCC 29212 was included as a quality-control strain in each run.

The expanded panel included representatives of major antimicrobial classes relevant to *Enterococcus* ecology and One Health surveillance (*β*-lactams, glycopeptides, oxazolidinones, macrolides, tetracyclines, phenicols, lincosamides, pleuromutilins, and fluoroquinolones); the full antimicrobial list and concentration ranges are provided in Supplementary Table S1.

MICs were interpreted using a dual framework: Clinical and Laboratory Standards Institute (CLSI) clinical categories (S/I/R), where species–drug clinical breakpoints were available, and European Committee on Antimicrobial Susceptibility Testing (EUCAST) epidemiological cut-off values (ECOFFs) to classify isolates as wild-type (WT) versus non-wild-type (NWT), where applicable. ECOFF-based categorization was treated as a surveillance tool (mechanism-oriented) rather than a clinical outcome predictor. This distinction was maintained throughout to ensure transparent interpretation when CLSI and EUCAST frameworks yield non-identical categorizations. Where EUCAST ECOFFs are species-restricted (e.g., linezolid ECOFFs for *E. faecalis* and *E. faecium*), WT/NWT classification was applied only to those species, while other *Enterococcus* spp. were interpreted using CLSI clinical categories and descriptive MIC distributions. For descriptive glycopeptide context in the enriched dataset, vancomycin resistance was operationally defined as MIC ≥ 4 μg/mL, and vancomycin summary outputs are provided in Supplementary Table S3 (phenotypic panel) and Supplementary Table S6 (WGS subset).

MDR status was assigned using a panel-based operational definition: non-susceptibility to at least one agent in ≥3 antimicrobial classes represented in the tested panel, following the standard harmonized terminology (34). Because MDR assignment depends on the tested antimicrobial classes, all MDR or extensively drug-resistant (XDR) descriptors are interpreted strictly within the scope of the applied panel.

### 2.3 Selection of isolates for whole-genome sequencing

Whole-genome sequencing (WGS) was performed on a targeted subset (*n* = 73) selected from the phenotyped MDR collection to maximize interpretability for the linezolid-focused objectives. Selection was enriched toward elevated/borderline linezolid MIC values, while maintaining representation across host species (chicken vs. turkey) and geographic regions. This WGS subset was therefore designed for mechanistic genotype–phenotype interrogation, not for prevalence inference.

Genomic DNA was extracted using the Zymo Quick-DNA Fecal/Soil Microbe Miniprep Kit (Zymo Research, Irvine, CA, United States) following the manufacturer’s instructions. DNA concentration and purity were assessed prior to library preparation. Libraries were prepared using the Nextera XT DNA Library Preparation Kit (Illumina, San Diego, CA, United States) and sequenced on an Illumina NextSeq 500 platform using paired-end chemistry.

### 2.4 Bioinformatic processing and genome reconstruction

Raw reads were quality-checked with FastQC v0.11.9 (35) and processed with fastp v0.23.2–3 (36). Error correction was performed with Bloocoo v1.0.7 (37). Adapter and quality trimming were carried out with TrimGalore v0.6.6 (38).

*De novo* assembly was performed using MEGAHIT v1.2.9 (39) and SPAdes v4.0.0 (40). Assembly reconciliation was performed with GAM-NGS v1.1b (41). Assemblies were evaluated with QUAST v5.2.0 (42) and completeness was assessed with BUSCO v5 (43). Contigs shorter than 250 bp were excluded from downstream analyses.

Protein-coding genes were predicted with Prodigal v2.6.3 (44). Acquired antimicrobial resistance genes were identified using the Resistance Gene Identifier (RGI) with CARD (45, 46), and cross-validated using two independent resources: ABRicate screening (47) against the ResFinder database and NCBI AMRFinderPlus. For oxazolidinone-associated determinants [*optrA, poxtA, cfr/cfr(B)*] we required high-confidence matches (≥95% nucleotide identity and ≥95% query coverage) and performed screening on assembled contigs (*de novo* assemblies from Illumina short reads). All database screening outputs are reported transparently in Supplementary Table S2. As a pipeline sanity check, the same workflow recovered *optrA* from an external positive-control *Enterococcus* assembly (GenBank CP041776.2) under the same thresholds.

To contextualize linezolid MIC phenotypes, genomes were additionally evaluated for canonical target-site mechanisms in *Enterococcus*, including variation in 23S rRNA and ribosomal protein genes [*rplC* (L3), *rplD* (L4), *rplV* (L22)]. The 23S rRNA G2576T substitution (*E. coli* numbering convention) was specifically assessed alongside other previously described substitutions relevant to oxazolidinone non-susceptibility.

Mobile genetic elements were screened using MobileElementFinder v1.0.3 (48). For transposon-associated assignments, a distance threshold of 10 kb was applied to support co-localization inferences. Putative plasmid-derived contigs were predicted using PlasFlow v1.1 (49). Viral sequence detection was performed using VirSorter2 v2.2.2 (50); only contigs ≥10 kb were considered for confident viral sequence reporting. Taxonomic assignment and genome quality checks were supported by CheckM v1.2.2 (51) and Kraken v1.1.1 (52).

*In silico* MLST was performed using species-specific PubMLST *Enterococcus* schemes (the *E. faecalis* scheme: *aroE–gdh–gki–gyd–pstS–xpt–yqiL*; and the *E. faecium* scheme: *adk–atpA–ddl–gdh–purK–gyd–pstS*). Sequence types (STs) were assigned by exact 7-locus allele-profile matching against the PubMLST *Enterococcus* databases (accessed April 09, 2026) using assembled contigs. STs were assigned only when all seven loci were recovered at 100% identity and 100% coverage; assemblies with incomplete profiles are reported as partial allele calls (no ST assigned).

## 3 Results

### 3.1 Collection overview and resistance-enrichment strategy

A previously established national *Enterococcus* isolate collection (*N* = 969), generated through routine farm surveillance and diagnostic workflows prior to the present study, served as the source material for all analyses. No animals were handled or sampled specifically for this study; only archived bacterial isolates and associated metadata were used. From this existing collection, after resistance-oriented pre-screening, 218 isolates were selected for broth microdilution MIC testing in an expanded panel that included linezolid. Selection was MDR-oriented rather than linezolid-only, which is reflected by the broad linezolid MIC range and the substantial susceptible fraction in the enriched panel. Because the workflow intentionally enriched for resistant phenotypes, all downstream proportions reflect an “enriched subset” and must not be interpreted as population prevalence.

### 3.2 Linezolid MIC distribution and transparent interpretation

Across the enriched phenotypic panel (*n* = 218), linezolid MICs ranged from 0.015 to 512 μg/mL (tested range: 0.015–1,024 μg/mL), with MIC_50_ = 2 μg/mL and MIC_90_ = 128 μg/mL (Table 1). The distribution was right-tailed, with a dominant mode at 2 μg/mL. The full MIC distribution and CLSI category overlay are shown in Figure 2.

**Table 1:** Linezolid minimum inhibitory concentration (MIC) distribution in the enriched phenotypic panel (*n* = 218).

Linezolid MIC (μg/mL)	*n*	%
0.015	2	0.9
0.5	3	1.4
1	23	10.6
2	111	50.9
4	37	17.0
8	2	0.9
16	5	2.2
32	7	3.2
64	5	2.3
128	6	2.8
256	13	6.0
512	4	1.8

Using an operational elevated-MIC cutoff of 4 μg/mL (aligned with the EUCAST linezolid ECOFF defined for *E. faecalis* and *E. faecium,* WT ≤ 4 μg/mL; NWT > 4 μg/mL for *E. faecalis and E. faecium*), 42/218 (19.3%) isolates showed MICs >4 μg/mL. Formal EUCAST WT/NWT categorization was applied only where ECOFFs are available (i.e., *E. faecalis* and *E. faecium*), and other *Enterococcus* spp. are reported as MIC distributions without ECOFF-based WT/NWT labeling.

Using CLSI breakpoints for *Enterococcus* (S ≤ 2 μg/mL; I = 4 μg/mL; R ≥ 8 μg/mL), 139/218 (63.7%) isolates were susceptible, 37/218 (17.0%) intermediate, and 42/218 (19.3%) resistant. Thus, the CLSI non-susceptible fraction (I + R) was 79/218 (36.2%). Notably, CLSI non-susceptibility (I + R) and EUCAST NWT address different questions and can yield different fractions in enriched datasets.

In a species-stratified exploratory analysis of MALDI-identified isolates, *E. faecium* showed higher linezolid MICs than *E. faecalis* (MIC_50_: 4 vs. 2 μg/mL; MIC_90_: 128 vs. 4 μg/mL), with a higher CLSI non-susceptibility fraction in *E. faecium* (9/16) than in *E. faecalis* (3/14) (Supplementary Figure S1). Given the targeted enrichment design and limited typed subgroup size in the phenotypic panel, these comparisons are interpreted descriptively.

### 3.3 Multidrug resistance burden in the enriched phenotypic panel

Applying panel-based MDR definitions (i.e., limited to the antimicrobial categories tested), 196/218 isolates (89.9%) met the MDR criterion. Among these MDR isolates (*n* = 196), resistance breadth spanned 3 to 9 antimicrobial categories, indicating extensive co-resistance within the enriched subset (Table 2). Notably, 16/196 (8.2%) MDR isolates were non-susceptible to ≥7 categories, including one isolate non-susceptible to all 9 tested categories (panel-limited interpretation).

**Table 2:** Resistance breadth among multidrug-resistant (MDR) isolates (*n* = 196).

No. of antimicrobial categories with non-susceptibility	*n*	%
3	34	17.3
4	54	27.5
5	55	28.1
6	37	18.9
7	7	3.6
8	8	4.1
9	1	0.5

To provide context on the MDR background of the enriched panel, we additionally summarized non-susceptibility frequencies by antimicrobial class (panel-limited interpretation) and provide the detailed class-level breakdown in Supplementary Table S3.

Antimicrobials with limited or no intrinsic activity against enterococci and/or lacking accepted interpretive criteria in this context (e.g., ceftriaxone, colistin, and neomycin) were not used for MDR classification or mechanistic inference. Where measured, these MIC distributions are provided descriptively in Supplementary material for completeness.

To make the MDR background explicit, we summarized the most frequent class-level MDR patterns within the enriched phenotypic panel (panel-limited interpretation). The dominant patterns reflected concurrent non-susceptibility across macrolides, tetracyclines, phenicols, and lincosamides, with additional contributions from *β*-lactams and/or fluoroquinolones in a substantial fraction of isolates (Supplementary Table S4).

### 3.4 Vancomycin MIC distribution and genomic context

In the resistance-enriched phenotypic panel (*n* = 218), vancomycin MICs showed an elevated tail. Using an operational study definition of vancomycin resistance (MIC ≥ 4 μg/mL), 76/218 (34.9%) isolates met this criterion, with MIC_50_ = 1 μg/mL and MIC_90_ = 256 μg/mL (Supplementary Table S3). These values are reported descriptively to contextualize glycopeptide signals captured under the enrichment design and are not intended for prevalence inference. In the WGS subset (*n* = 73), vancomycin MICs ranged from 0.5 to 512 μg/mL, and 35/73 (47.9%) isolates had MICs ≥ 4 μg/mL (study definition) (Supplementary Table S6). Screening of the sequenced subset did not identify high-confidence acquired vancomycin resistance operons (*vanA/vanB/vanD/vanM*). High-confidence *vanC*-cluster signatures were detected in four genomes (Isolate IDs: 142, 198, 322, 444), and a single low-coverage *vanW* fragment was observed in isolate 198; these findings are reported for context without implying validated transfer.

### 3.5 Whole-genome sequenced subset and taxonomic composition

Whole-genome sequencing was performed on an MDR-enriched subset (*n* = 73), selected to over-represent elevated/borderline linezolid MICs and stratified across poultry source and geography (enrichment design; not prevalence). Species composition of the WGS subset is shown in Table 3. WGS-based taxonomic assignment (Kraken-supported) was concordant with MALDI-TOF identifications for the sequenced subset, including the non-*faecalis/faecium Enterococcus* species. MLST identified multiple distinct allele profiles within both *E. faecium* and *E. faecalis* in the sequenced subset (Supplementary Table S5), supporting that the observed linezolid non-susceptibility signals were not restricted to a single shared 7-locus profile. We report these outputs for context and do not use MLST alone to infer clonal spread (Supplementary Table S5).

**Table 3:** Species distribution of the whole-genome sequencing (WGS) subset (*n* = 73).

Species	*n*	%
*Enterococcus faecalis*	44	60.3
*Enterococcus faecium*	26	35.6
*Enterococcus casseliflavus*	2	2.7
*Enterococcus gallinarum*	1	1.4

**Figure 3:**
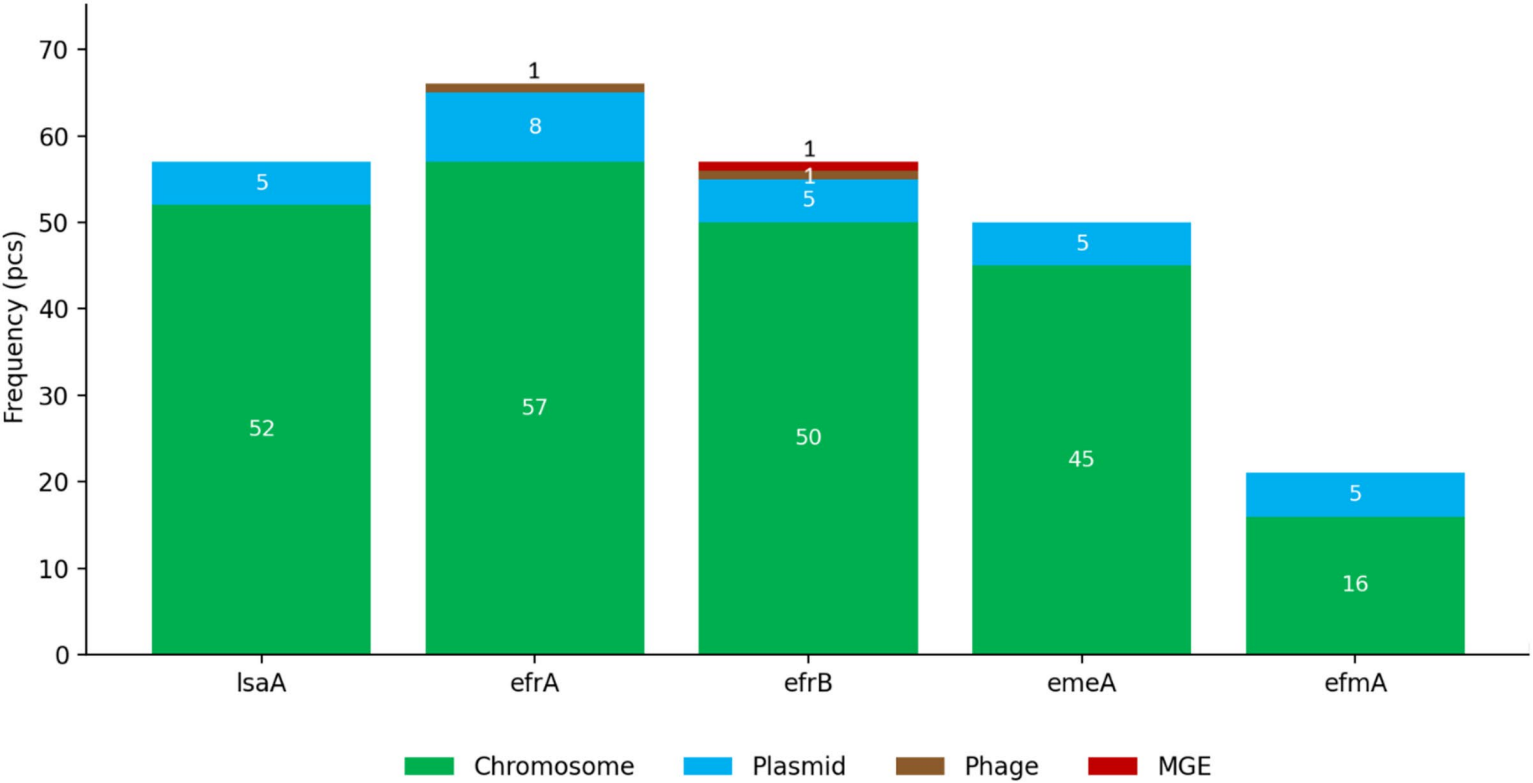
Predicted genomic localization of selected efflux- and resistance-associated loci in the sequenced subset (*n* = 73). Stacked bars show, for each locus, the number of genomes in which the gene was detected and its *in silico* predicted genomic context (chromosome, plasmid-associated contig, phage-associated contig, or other mobile genetic element). Context assignments are based on computational prediction and do not imply experimental validation of mobility or transfer. Gene names are shown as detected in the whole-genome sequencing (WGS) dataset.

Across the 73 sequenced genomes (Table 4), no canonical transferable oxazolidinone resistance determinants were detected [*optrA, poxtA, cfr/cfr(B)* all absent]. To enable direct phenotype–genotype overview, isolate-level linezolid MIC values are reported alongside key oxazolidinone determinants and selected context markers for the WGS subset in Supplementary Table S6. The absence of *optrA/poxtA/cfr* was concordant across CARD/RGI, ResFinder, and AMRFinderPlus screenings (Supplementary Table S7). In contrast, loci annotated in resistance databases as potentially associated with oxazolidinone/MLS_B–related phenotypes or efflux-based modulation were frequent, led by *lsaA* (57/73; 78.1%) and the efflux-associated *efrA* (66/73; 90.4%), *efrB* (57/73; 78.1%). To contextualize alternative genomic features in the absence of canonical transferable oxazolidinone determinants, we summarized the predicted genomic localization of selected efflux- and resistance-associated loci across the sequenced subset; these loci were most often detected on contigs without plasmid/phage/MGE signatures; however, all context assignments are contig-level computational predictions based on short reads and should not be interpreted as evidence of mobility or transfer (Figure 3).

**Table 4:** Whole-genome sequencing (WGS) subset (*n* = 73), presence/absence of selected loci in the WGS subset (canonical oxazolidinone determinants and context markers).

Determinant	*n*	%
*optrA*	0	0
*poxtA*	0	0
*cfr*	0	0
*cfr(B)*	0	0
*lsaA*	57	78.1
*lsaE*	10	13.7
*msrC*	13	17.8
*efrA*	66	90.4
*efrB*	57	78.1
*emeA*	50	68.5
*efmA*	21	28.8

Critically, a 23S rRNA G2576T substitution (*E. coli* numbering convention) was identified in a subset of genomes (7 isolates), consistent with a target-site mechanism that can underlie clinically relevant linezolid resistance even in the absence of known transferable determinants. Among these seven isolates, linezolid MICs were 256**–**512 μg/mL (median 256 μg/mL), and the mutation occurred in species: *E. faecalis* (5/7) and *E. faecium* (2/7) (Supplementary Table S8). This observation is handled as chromosomal/target-mediated risk, distinct from horizontal-transfer risk, and is reported without over-claiming phenotype causality beyond the available validation. No additional recurrent nonsynonymous variants were detected in rplC (L3), rplD (L4), or rplV (L22) that consistently segregated with elevated linezolid MICs in the sequenced subset.

## 4 Discussion

Linezolid is a critical agent for the treatment of severe infections caused by MDR Gram-positive bacteria, and the emergence of linezolid non-susceptibility outside the human clinical setting raises clear One Health concerns across the food chain, animal reservoirs, and the environment (6–8, 53). *Enterococcus* spp. are particularly relevant sentinels in this context because they are ubiquitous in the gastrointestinal tract of animals and humans, persist well in diverse environments, and combine ecological fitness with substantial adaptive capacity (10, 11, 13, 15). Moreover, *E. faecalis* and *E. faecium* are clinically important opportunistic pathogens, and their ability to accumulate resistance determinants and persist under strong selection pressure underlines why resistance signals emerging in non-clinical reservoirs deserve careful, mechanism-oriented interpretation (19, 20, 54, 55).

A key strength of the present work is the explicit separation of clinical categorization and epidemiological (WT/NWT) classification when interpreting linezolid MIC distributions. These frameworks address different questions: clinical breakpoints support therapy-oriented categories, while ECOFF-based WT/NWT classification is intended to flag acquired resistance mechanisms at the population level (30). This distinction is not academic: borderline MIC values may be categorized differently depending on the applied interpretive scheme, which can complicate cross-study comparability unless the framework is stated transparently (30). Reporting both interpretations therefore strengthens reproducibility and reduces ambiguity in One Health surveillance where clinical outcome inference is not the primary aim (6–8).

From a genomic perspective, the most consequential observation is that the sequenced MDR subset did not harbor the major transferable oxazolidinone resistance determinants commonly discussed in *Enterococcus,* most notably the *cfr* family and the ABC-F ribosomal protection genes *optrA* and *poxtA* (21, 56–58). This negative finding is important, because multiple studies have described plasmid-associated *cfr* carriage and emphasized the potential for horizontal dissemination of oxazolidinone resistance, while *optrA* and *poxtA* have been repeatedly reported in *Enterococcus* from animal, food-chain, and environmental contexts (21). In practical terms, our data suggest that, within this Hungarian poultry MDR subset, elevated linezolid MICs are unlikely to be driven primarily by these canonical transferable determinants, highlighting that the oxazolidinone resistance “risk profile” may differ substantially between settings and surveillance frames (57, 58).

In contrast to the absence of the above transferable genes, we detected a 23S rRNA target-site substitution (G2576T) in a subset of isolates, and this signal coincided with high-level phenotypes in our dataset. Target-site mutations in 23S rRNA are a well-established route to linezolid resistance in enterococci and represent a mechanistic category distinct from mobile gene-mediated resistance (26). This distinction is One Health-relevant: the absence of transferable determinants does not equate to the absence of risk, because chromosomal resistance can still enable persistence and clonal expansion of resistant lineages if ecological or clinical conditions permit (19, 26). For the dominant loci (*lsaA, efrA, efrB*), the detected sequences showed high similarity to reference database entries based on hit-level identity and coverage metrics, and we did not observe systematic divergence patterns suggestive of a novel allelic variant driving linezolid MIC elevation; where partial alignments occurred, they were consistent with contig fragmentation in short-read assemblies (Supplementary Table S9).

We also observed that not all elevated or borderline MIC phenotypes can be straightforwardly explained by the currently best-characterized oxazolidinone determinants. This phenotype–genotype gap should be handled conservatively: it may reflect additional or rare target-site variants, ribosomal protein alterations, or broader MDR-associated physiology affecting drug accumulation rather than a single identifiable mobile determinant (21, 59). Importantly, the frequent detection of efflux- and stress-response-associated loci in MDR enterococci should be interpreted as context rather than direct causality for linezolid resistance unless experimentally validated, particularly when these loci are predominantly chromosomal and not oxazolidinone-specific predictors (55, 59).

Several limitations should be emphasized to avoid overreach. First, the phenotypic dataset derives from a resistance-enriched subset and is not designed for prevalence inference. Second, interpretive systems were developed primarily for clinical decision-making and surveillance in human medicine; applying them to animal-derived isolates requires explicit framing and careful wording (60). Third, WGS was performed on a targeted subset selected to resolve the linezolid-focused question and does not represent the full diversity of the original collection. Together, these limitations underscore why One Health surveillance benefits most from integrated designs that pair MIC distributions with genome-resolved mechanism screening and that clearly separate transferable-gene risk from target-mediated resistance dynamics (6–8, 26, 56). Importantly, while G2576T explained high-level linezolid MICs in a subset, a portion of elevated/borderline phenotypes remained under-resolved by currently canonical determinants, which we interpret as a conservative genotype–phenotype gap rather than evidence for a specific alternative mechanism. Accordingly, we avoid overinterpreting “emergence” trajectories and present the dataset as a surveillance-oriented foundation motivating follow-up work (e.g., deeper variant analysis, copy-level resolution, and expression-level assays). A similar genotype–phenotype gap was observed for vancomycin. Although the enrichment design captured an elevated vancomycin MIC tail, no high-confidence acquired *vanA/vanB*-type operons were detected in the sequenced subset. Accordingly, we report the vancomycin signal descriptively and interpret it conservatively as phenotype–genotype discordance that motivates follow-up, rather than as definitive evidence of widespread acquired glycopeptide resistance.

## 5 Conclusion

In conclusion, this study provides a phenotypic–genomic characterization of linezolid non-susceptibility in poultry-derived multidrug-resistant *Enterococcus* spp. from Hungary under a resistance-enriched surveillance design. The linezolid MIC distributions revealed a non-trivial fraction of non-susceptible phenotypes within the enriched subset, while targeted whole-genome sequencing showed that these phenotypes were not driven by canonical transferable oxazolidinone resistance genes. Instead, we identified a target-site mechanism in a subset of isolates consistent with high-level resistance, underscoring that clinically relevant oxazolidinone non-susceptibility can emerge and persist even in the absence of recognized mobile determinants. Importantly, the enrichment-based design precludes prevalence inference, but it enables mechanistic resolution of elevated MICs and highlights interpretive pitfalls when clinical categories and epidemiological WT/NWT classification are conflated. Taken together, our approach offers a practical template for One Health surveillance that integrates MIC distributions with genome-informed screening to distinguish mobility-associated risk from chromosomal pathways, thereby supporting more precise monitoring, early warning, and risk assessment across the human–animal interface.

## Data Availability

The datasets used and/or analyzed during the current study are available from the corresponding author upon reasonable request. Assembled genome contigs have been deposited in the NCBI BioProject database under accession PRJNA1395003.
