# Supplementary figures and images for: Phenotypic and genomic characterization of linezolid non-susceptibility in poultry-derived multidrug-resistant Enterococcus spp. from Hungary
Source: Front Vet Sci. 2026 May 19;13:1781468. doi: 10.3389/fvets.2026.1781468 (PMC13226017; doi:10.3389/fvets.2026.1781468)

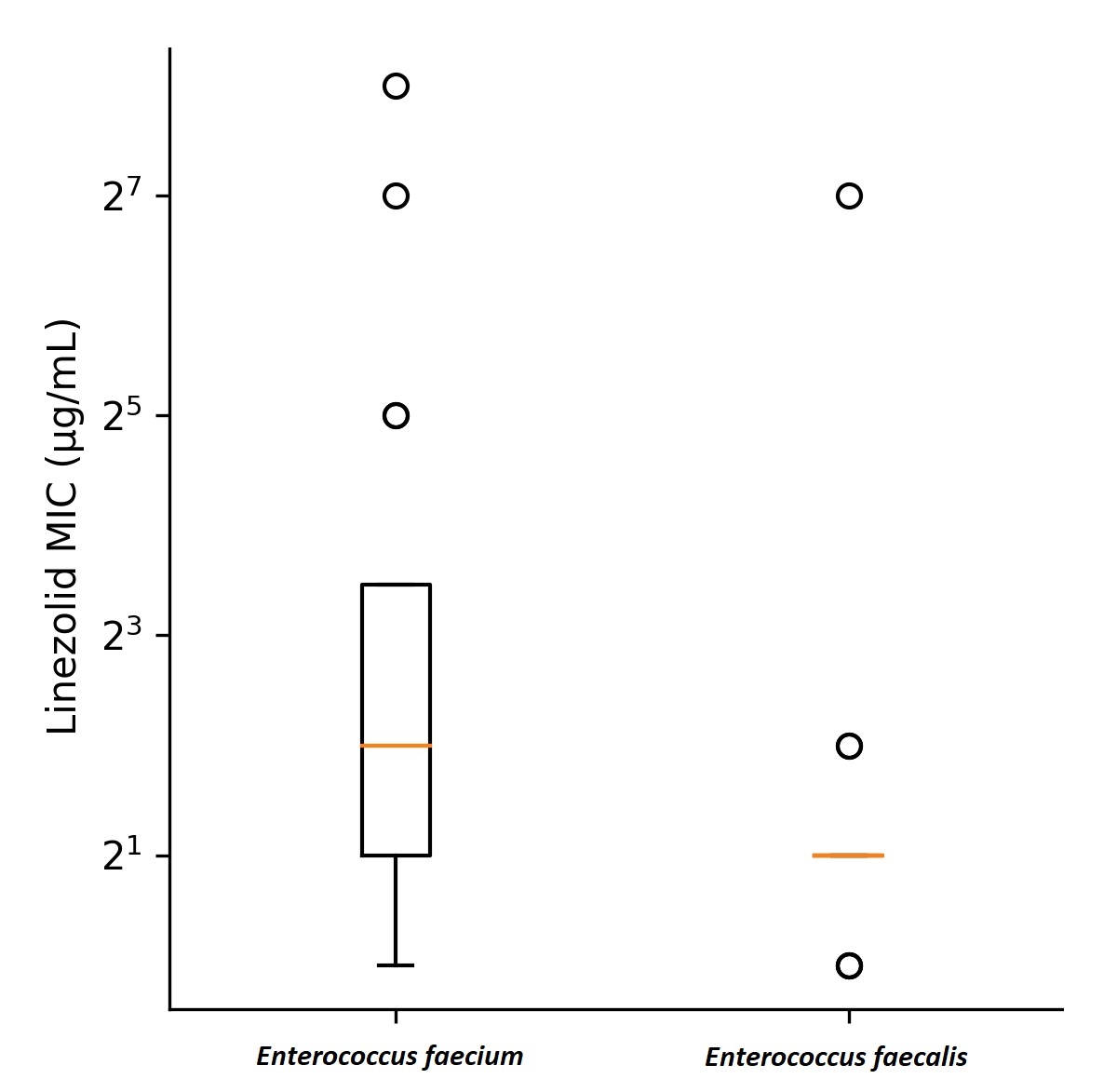

Supplement: Supplementary file 1 [file Image_1.jpeg]
